# The use of accelerometers to assess upper limb function in patients with obstetric brachial plexus palsy

**DOI:** 10.1038/s41598-024-72845-7

**Published:** 2024-09-27

**Authors:** Tim Leypold, Jörg Bahm, Benedikt Schäfer, Justus P. Beier, Catherine Disselhorst-Klug, Ligia C. Fonseca-Höflinger

**Affiliations:** 1 Department of Plastic Surgery, Hand Surgery-Burn Center, University Hospital RWTH Aachen, Pauwelsstraße 30, 52074 Aachen, Germany; 2 Department of Rehabilitation & Prevention Engineering, Institute of Applied Medical Engineering, RWTH Aachen University, Pauwelsstraße 20, 52074 Aachen, Germany; 3 Division for Plexus Surgery, Department of Plastic Surgery, Hand Surgery – Burn Center, University Hospital RWTH Aachen, Pauwelsstraße 30, 52074 Aachen, Germany

**Keywords:** Accelerometers, Plastic surgery, Brachial plexus palsy, OBPP, Movement analysis, Sensors, Paediatric research, Engineering, Neurological disorders

## Abstract

For half a century, the Mallet Scale (MS) has been utilized to assess upper limb function in patients with obstetric brachial plexus palsy (OBPP). However, the correct use of the MS requires trained personnel and the MS does not measure compensatory movements. For this reason, new methods are needed to compensate for these weaknesses. This study introduces an innovative method for objective functional motion analysis using accelerometers to measure upper limb movements in thirty patients with obstetric brachial plexus lesions. Five triaxial accelerometers were positioned on the chest and each upper limb. They recorded acceleration signals during repetitive everyday tasks: hand-to-mouth (HM), hand-to-neck (HN), and hand-to-spine (HS). From these signals, 54 features were extracted and subjected to linear correlation tests to identify 5 suitable features. An algorithm was then developed to categorize patients into five groups and compute an individual movement performance score (iMPScore) assessing the patient’s upper extremity function. By using the iMPScore more than 75% of all participants have been classified correctly with respect to their MS category. Identification of MS I category patients in general and assessing upper extremity function of MS I to III in HS tasks were most challenging. We conclude that the introduced approach is a valuable tool for gauging movement limitation of upper limbs in patients with obstetric brachial plexus palsy. Compared to other clinically established methods, it becomes possible to record and even quantify the extent of compensatory movements. In this way, an objective, user- and patient-friendly method is offered, which supports significantly physicians and therapists in their evaluation of OBPP.

## Introduction

The obstetric brachial plexus palsy (OBPP) is an uncommon birth trauma that affects the nerve network originating from spinal roots C5-T1, with an incidence rate of about 1.3 to 1.5 in every 1000 newborns. The severity of arm function impairment varies, with at least 20% of cases showing permanent damage^1^.

Patients’ coping strategies are highly individualized due to the unique location, extent, and severity of each brachial plexus lesion. Regular assessment of upper limb function is crucial to monitor natural improvement, establish treatment goals and follow postoperative improvement^2^.

Currently, this is primarily achieved through visual observations by physicians or therapists, supplemented by various rating scales such as the Gilbert and Raimondi scale for the elbow, the Raimondi Hand Score for the hand and the Mallet Scale (MS) for the shoulder^2–4^.

The MS includes five distinct shoulder movements, each designed to evaluate different aspects of shoulder mobility and function:

1. Active Abduction: This movement assesses the patient’s ability to lift the arm sideways away from the body.
2. External Rotation: This measures the ability to rotate the arm outward, away from the center of the body.
3. Hand-to-Mouth: This tests the patient’s capability to bring their hand to their mouth, simulating the action of eating.
4. Hand-to-Neck: This evaluates the ability to touch the back of the neck, which involves both abduction and external rotation.
5. Hand-to-Spine: This assesses the ability to reach behind the back to touch the spine, testing internal rotation and extension.

Each movement is graded on a scale from I to V:

- Grade I indicates minimal or no ability to perform the movement (frail shoulder).
- Grade II shows partial movement with significant difficulty.
- Grade III indicates fair movement with moderate difficulty.
- Grade IV shows good movement with mild difficulty.
- Grade V represents normal movement without difficulty.

By providing a detailed breakdown of these movements, the MS offers a comprehensive assessment of shoulder function, enabling targeted interventions and tracking of patient progress^4^.

The World Health Organization studied function more in detail and came to a categorization into three domains, developed within the International Classification of Function, Disability and Health (ICF). These domains concern body functions and structures, activity, and participation^5^. Recent scales like the Brachial Plexus Outcome Measure (BPOM) assess these domains^6^. However, all currently available assessment scores have common weaknesses, like interobserver variability, problems with patient cooperation (compliance), lack of standardization across clinics and poor information about compensational movements^7,8^. A survey conducted by Shin et al. revealed significant methodological differences among expert brachial plexus surgeons when measuring patient outcomes^8^. Furthermore, patients’ functional abilities in clinical settings often differ from their everyday life performance^9^.

Technological advancements like three-dimensional (3D) movement analysis offer objective analysis of limb movement^10^. While lower limb movement analysis is well-established (dealing with a repetitive motion pattern in gait), upper extremity analysis is more complex due to the nature of free arm movement and lack of standardized data analysis procedures. Moreover, 3D motion analysis procedures are technically complex and costly^10,11^.

Accelerometers offer a promising new tool for recording upper limb movement performance. They are non-invasive, compact with very little equipment, affordable, and provide objective motion observation^12^. By quantifying the dynamic characteristics of movements, specifically the execution speed and its first derivative, acceleration, they provide valuable data. The use of accelerometers is simple and ensures that the methodology is easily tolerated by children, who may find complex measurement procedures challenging or infeasible. In perspective, accelerometers enable movement analysis in everyday life^13^, which is not possible with previous measurement methods and assessments. It is consistent with the domains of the ICF and could be considered the ideal instrumental measurement not only of body functions and structures, but also for activity and participation^7^.

Our study combines accelerometers with a classification procedure for quantitative assessment of upper extremity movement performance in OBPP patients. The MS, widely used to measure shoulder motion impairment associated with OBPP^4,14–16^ was used on a comparative basis. MS categorizes upper limb and especially shoulder dysfunction into five severity levels. While it does not consider coping strategies, our approach specifically identifies and incorporates postural coping strategies.

The primary hypothesis of this study is that accelerometer signal classification is consistent with the MS clinical test results in patients with OBPP. The goal is to determine if accelerometers can effectively capture the nuances of upper limb movement quality in OBPP patients.

Furthermore, this study aims to validate the use of accelerometers as an objective tool for assessing upper extremity function, potentially offering a more standardized and quantifiable approach compared to traditional clinical observations. By comparing accelerometer data with the MS results, the study seeks to explore the potential of accelerometry in improving the accuracy and reliability of patient evaluations.

As a preliminary study, this research also intends to lay the groundwork for future studies that extend the use of accelerometers to measure everyday life activities. This future direction aims to enhance the applicability of accelerometry in real-world settings, providing continuous and comprehensive monitoring of patients’ functional improvements over time.

## Materials and methods

### Subjects

20 OBPP patients (MS IV – I) aged 11.9 ± 5.3 years were recruited during medical consultations at the Division of Plexus Surgery, Department of Plastic Surgery, Hand Surgery – Burn Center, University Hospital RWTH Aachen, Germany. Additionally, 10 healthy children of the same age span were included in the study, representing MS V (= normal shoulder function).

The inclusion criteria for patients were as follows: clinical diagnosis of OBPP, informed consent by the parents / legal guardians, ability to perform the test protocol activities and an age over 5 years. Exclusion criteria were the inability to perform the proposed tasks and refusal to give informed consent.

The institutional ethics committee of our institution (EK254/16) granted approval for the study, and every participant’s parents / legal guardians gave their written informed consent prior to the evaluation.

### Test protocol

Prior to movement assessment, the procedures were explained to the subjects. Participants were asked to perform three simple tasks that are also included in the assessment of movement capacity using the MS^4,17^. These were hand-to-mouth (HM); hand-to-neck (HN) and hand-to-spine (HS). Each task was performed separately with both the right and left arm.

The execution of each task was evaluated by an experienced physiotherapist and categorized according to the MS. Afterwards, the subjects stood in neutral position with the arms alongside the body and the thumb pointing anterior. From this position, each task was repeated 12 times without a break in a self-determined velocity. The subjects attempted to perform two trials, each of 12 repetitions, with a one-second pause between them. The order in which the three tasks were performed was random, as was the arm with which the exercise test was started.

### Instrumentation

**Fig. 1:**
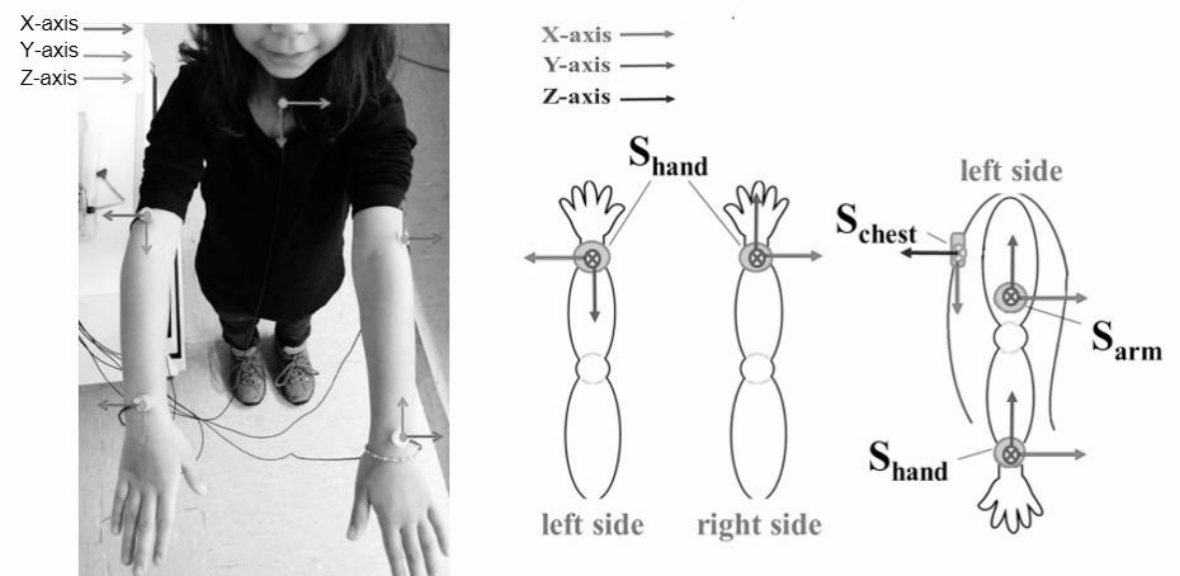
A total number of 5 triaxial accelerometers were placed on both arms and the chest in order to assess movement performance. Orientation of the sensors has been standardized.

To investigate movement performance of both the affected and the unaffected arm during the tasks, a total of 5 triaxial ultra-high-performance g-sensor accelerometers (BMA 180, Robert Bosch GmbH, Germany) with 14-bit ADC operation have been used. Sensitivity of the accelerometers ranges from +/- 1 g to a maximum of +/- 16 g, depending on the g-range chosen. Four sensors were placed symmetrically on the left and right arm of the subjects. One sensor was attached to the wrist between the distal end of ulna and radius of the hands and one sensor on the distal part of the humerus of both arms. The fifth sensor was positioned at the manubrium of the sternum (Fig. 1).

To ensure that the cables did not hinder the movement of the arm, they were laid in a posterior direction (Fig. 1). In addition, it was ensured that the sensors were fixed in the same position on both arms. Double-sided adhesive tape was used to achieve a secure and robust fixation of the sensors on the skin.

### Data processing

Acceleration can be represented in Cartesian coordinates along the X-, Y- and Z-axis of each sensor as shown in Fig. 1. However, mathematically the same information can be represented in the spherical coordinates elevation (ϕ), azimuth (θ) and radius (r). In doing so, 6 acceleration signals, 3 in Cartesian coordinates and 3 in spherical coordinates, can be gained from each sensor. Considering one arm executing the task and three sensors (hand, arm, and chest) detecting the movement performed, this results in a total number of 18 acceleration signals for each task.

### Features describing the acceleration signals

Three different features (F_i_) were utilized to determine the difference between movements of the affected and the non-affected arm when performing the same task.

#### F1: difference in maximal acceleration (DiffMax)

The feature Diff_Max_ describes the difference between the maximal acceleration recorded from the affected and the non-affected arm. First, for each of the 12 repetitions of a movement task the maximal acceleration value was determined separately for each sensor signal. Afterwards the determined values have been averaged across all repetition. Finally, the mean maximal acceleration value of each signal determined from the un-affected limb have been subtracted from the corresponding mean maximum acceleration value of the affected limb.

$$\:\text{D}\text{i}\text{f}{\text{f}}_{\text{M}\text{a}\text{x}}=\text{M}\text{e}\text{a}\text{n}\left(\text{M}\text{a}{\text{x}}_{\text{a}\text{f}\text{f}\text{e}\text{c}\text{t}\text{e}\text{d}}\right)-\text{M}\text{e}\text{a}\text{n}\left(\text{M}\text{a}{\text{x}}_{\text{n}\text{o}\text{n}-\text{a}\text{f}\text{f}\text{e}\text{c}\text{t}\text{e}\text{d}}\right)$$

In the case of the chest sensor, the mean maximal acceleration recorded when the movement is performed with the non-affected arm has been subtracted from the mean maximal acceleration, recorded when the task is performed with the affected arm.

#### F2: cross-correlation coefficient (CCCoef)

Cross-correlation is a common method used in signal processing to determine the similarity between two signals. To assess the similarity, the two considered signals are shifted in time relative to each other and the area by which the signals overlap is determined for each shifted sample (m). This results in the cross-correlation function (CC(m)), which gets its maximum when highest similarity is reached. The maximum of the cross-correlation function is called cross-correlation coefficient (CC_Coef_) and is a measure of similarity. CC_Coef_ has a value of “+1” or “-1” if the signals are completely identical and “0” if there is no match. To determine the differences in movement performance between the affected arm and the non-affected arm, corresponding acceleration signals from both sides of the body were compared and CC_Coef_ was calculated.

$$\:{\text{C}\text{C}}_{\text{C}\text{o}\text{e}\text{f}}=\text{M}\text{a}\text{x}\left[\text{C}\text{C}\left(\text{m}\right)\right]=\text{M}\text{a}\text{x}\left[\frac{1}{\text{N}}\sum\:_{\text{n}=0}^{\text{N}-\text{m}-1}{\text{S}\text{i}\text{g}\text{n}\text{a}\text{l}\:\left(\text{n}\right)}_{\text{a}\text{f}\text{f}\text{e}\text{c}\text{t}\text{e}\text{d}}\text{*}{\text{S}\text{i}\text{g}\text{n}\text{a}\text{l}\:(\text{n}+\text{m})}_{\text{n}\text{o}\text{n}-\text{a}\text{f}\text{f}\text{e}\text{c}\text{t}\text{e}\text{d}}\right]\:$$

CC(m): Cross-correlation function; m: Number of samples shifted; N: Number of samples contributing to the signal.

#### F3: area outside the standard deviation (Areaout)

The feature “Area outside the standard deviation (Area_out_)” is a measure of the probability that the signal detected from the affected arm differs from that gained from the non-affected arm. To calculate this feature, signal segments according to a movement cycle of the non-affected arm are first identified and afterwards time-normalized. Identified time-normalized signal segments are averaged across all repetitions of the task and mean values (Mean) and standard deviations (Std) are determined. The same procedure is followed for the affected side. If the mean value of the affected side is either higher or lower than the mean value plus/minus its standard deviation of the non-affected side, the difference (Diff(n)) between both values is calculated. Subsequently, all calculated differences are then summed up in order to obtain an area measure (Area_out_) which describes the level of deviation of the movement of the affected arm from that of the non-affected arm.

$$\:{\text{A}\text{r}\text{e}\text{a}}_{\text{o}\text{u}\text{t}}=\:\frac{1}{\text{N}}\sum\:_{\text{n}=1}^{\text{N}}\text{D}\text{i}\text{f}\text{f}\left(\text{n}\right)\:\:\:\:\:\:\:\:\:\:\:\:$$

$$\:\text{D}\text{i}\text{f}\text{f}\left(\text{n}\right)=\left\{\left.\begin{array}{c}{\text{M}\text{e}\text{a}\text{n}}_{\text{a}\text{f}\text{f}\text{e}\text{c}\text{t}\text{e}\text{d}}-{\left(\text{M}\text{e}\text{a}\text{n}+\text{S}\text{t}\text{d}\right)}_{\text{n}\text{o}\text{n}-\text{a}\text{f}\text{f}\text{e}\text{c}\text{t}\text{e}\text{d}},\:if\:\:{\text{M}\text{e}\text{a}\text{n}}_{\text{a}\text{f}\text{f}\text{e}\text{c}\text{t}\text{e}\text{d}}>{(\text{M}\text{e}\text{a}\text{n}+\text{S}\text{t}\text{d})}_{\text{n}\text{o}\text{n}-\text{a}\text{f}\text{f}\text{e}\text{c}\text{t}\text{e}\text{d}}\\\:{\left(\text{M}\text{e}\text{a}\text{n}+\text{S}\text{t}\text{d}\right)}_{\text{n}\text{o}\text{n}-\text{a}\text{f}\text{f}\text{e}\text{c}\text{t}\text{e}\text{d}}-{\text{M}\text{e}\text{a}\text{n}}_{\text{a}\text{f}\text{f}\text{e}\text{c}\text{t}\text{e}\text{d}},\:if\:\:{\text{M}\text{e}\text{a}\text{n}}_{\text{a}\text{f}\text{f}\text{e}\text{c}\text{t}\text{e}\text{d}}<{(\text{M}\text{e}\text{a}\text{n}-\text{S}\text{t}\text{d})}_{\text{n}\text{o}\text{n}-\text{a}\text{f}\text{f}\text{e}\text{c}\text{t}\text{e}\text{d}}\end{array}\right\}\right.$$

N: Number of samples contributing to the time-normalized signal representing a movement cycle.

The values of the three individual features were calculated for each participant. In the case of healthy subjects, the dominant side was considered as non-affected and the non-dominant side as affected.

### Identification of relevant features for each movement task

**Fig. 2:**
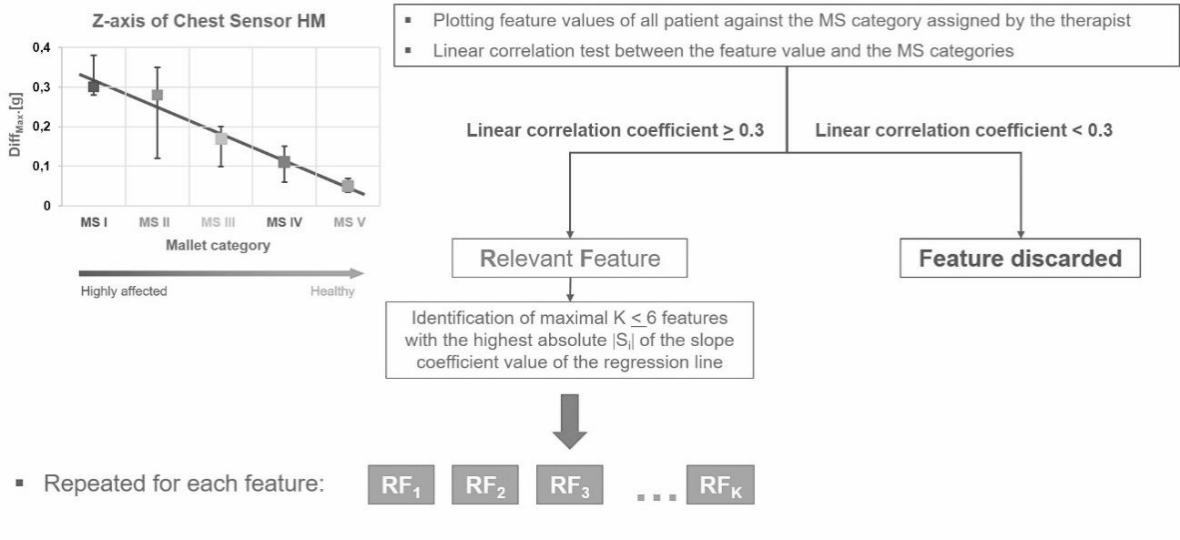
Procedure for identifying the relevant features (RF) for each movement task. Left side: Example of the hand-to-mouth task (HM) and the Diff_Max_ feature calculated from the Z-axis of the chest sensor. The process results in a set of maximal K ≤ 6 relevant features that best distinguish between the subjects of the respective MS category.

Regarding the three features calculated from each of the 18 acceleration channels, a total number of 54 features can be determined (3 features * [3 signals in Cartesian coordinates + 3 signals in spherical coordinates] * 3 sensors) for each movement task. As this number is far too high for a reliable statistical analysis, for each task those features need to be identified which achieve the best possible discrimination between patients in the individual categories of MS. To identify the relevant features of each task, a linear correlation test between the feature values and the MS categories has been applied. Figure 2 shows the procedure exemplarily for the Diff_Max_ feature calculated from the Z-axis of the Chest Sensor during the performance of the hand-to-mouth (HM) task.

First, all subjects were grouped according to the MS category assigned to by the therapist. Then, the feature value of each subject was plotted against its MS category (Fig. 2 on the left). Afterwards, a linear regression test was performed on the data and the linear correlation coefficient (R^2^) as well as the slope of the regression (S) were determined. This procedure was repeated for each feature.

Only those features with a linear correlation coefficient (R^2^) equal to or above 0.3 were considered as relevant features. Features with a correlation coefficient below 0.3 were discarded. As the number of features may not exceed the square root of the total number of participating subjects, the number of features was limited to a maximum of 6 to achieve statistically relevant results. For this purpose, a maximum of 6 features with the highest dependency of the feature on the MS category represented by the highest absolute |S_i_| of the slope coefficient value of the regression line were selected from the previously identified ones. To its end, the process results in a set of maximal K ≤ 6 relevant features (RF_i_) that best distinguish between the subjects of the respective MS category. The relevant features (RF_i_) were determined separately for each movement task.

### Individual movement performance score (iMPScore) assessing the patient’s upper extremity function

**Fig. 3:**
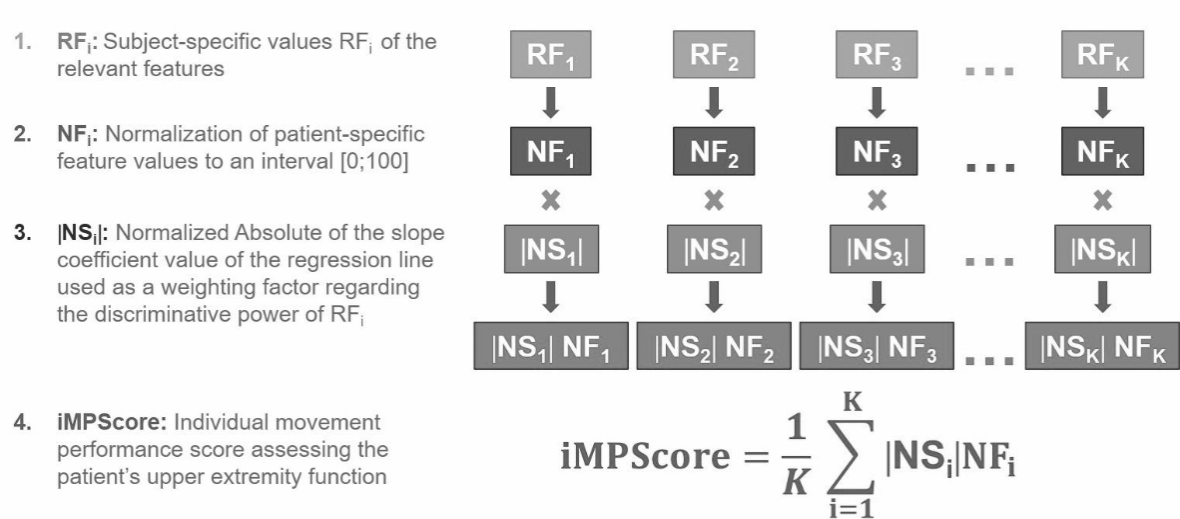
Representation of the single processing steps for calculating the individual movement performance score (iMPScore) based on the patient-specific values of the relevant features.

To assess the subjects’ individual upper extremity function quantitatively, the individual movement performance score (iMPScore) was introduced. The iMPScore was calculated from the subject-specific values of the relevant features (RF_i_) identified for each movement task (Fig. 3). Since the magnitude of the values of the different features differs and in order to prevent an effect on the iMPScore, in a first step RF_i_ values have been normalized to an interval from 0 to 100. For features in which a better movement performance is associated with higher feature values (positive slope of the regression line) 100 corresponds to the highest feature value that was found in healthy subjects. Features, in which a lower feature value is associated with higher movement performance (negative slope of the regression line), have first to be transformed in order to be able to normalize them in the same way as features with a positive slope of the regression line. Therefore, the feature values are first multiplied by -1, which corresponds to mirroring the regression line on the abscissa axis, resulting in the slope changing from negative to positive. Subsequently, the newly created line is shifted upwards by the value of the intersection of the regression line with the ordinate axis. 100 now corresponds to the highest transformed feature value present in healthy participants. The normalization process results in K ≤ 6 normalized relevant features (NF_i_), in which values close to 100 correspond to excellent movement performance while a value close to 0 means that almost no movement was possible (Fig. 3).

As not all features are equally predictive, the absolute |S_i_| of the slope coefficient of the regression line calculated after normalization of the features was used as a weighting factor, because the link between the feature values and the MS category is the stronger, the higher the slope of the regression line. To ensure that the iMPScore remains comparable between different movement tasks, the absolute slope coefficient was normalized |NS_i_|, with the maximal slope coefficient set to 1. Afterwards, to calculate the iMPScore normalized feature values (NF_i_) were first multiplied with |NS_i_|. Finally, summation of the weighted and normalized values of the relevant features leads to the individual movement performance score (iMPScore), which assesses the individual ability of each patient to perform the three movement tasks (Fig. 3).

$$\:\text{i}\text{M}\text{P}\text{S}\text{c}\text{o}\text{r}\text{e}=\frac{1}{K}\:\sum\:_{i=1}^{K}\left|{NS}_{i}\right|{NF}_{i}\:$$

K: Number of relevant features; NF_i_: Feature values normalized to an interval from 0 to 100; |NSi|: Normalized absolute of the slope coefficient of the regression line.

Due to the chosen normalization procedure, high values of the iMPScore represent excellent upper limb function, while lower values indicate poor movement performance.

### Statistical analysis

SPSS (SPSS Inc., Chicago, IL, USA) has been used for all statistical procedures. As only very few participants were assigned to some of the categories, a test for statistical significance between the feature values of participants assigned to different MS categories has been discarded. Instead, it was evaluated whether the assignment of a subject to a MS category by an experienced therapist could be confirmed by the iMPScore.

Unlike the MS, which only allows a subject to be assigned to one of the 5 categories, the iMPScore provides continuous values between 0 and 100. To compare the assignment of participants to an MS category with the iMPScore, a simple cluster analysis procedure was used with the aim of identifying 5 clusters, i.e. groups with similar iMPSores, in the group of participants. Hereby, each of the 5 clusters should be as homogeneous as possible, while the clusters should differ from each other as much as possible. To achieve this, a non-hierarchical cluster analysis based on the k-means algorithm was chosen. First, participants are randomly assigned to one of 5 clusters. Afterwards the median of all iMPScore values belonging to each cluster was calculated and a new assignment of participants to the clusters starts based on the smallest difference between the cluster’s median value and the individual iMPScore. Then, the median value is again calculated for each of the newly assembled 5 clusters. This iterative procedure is repeated until the composition of the clusters did not change significantly anymore. Finally, 5 clusters (A = high iMPScores, B, C, D, E = low iMPScores) have been identified as well as threshold for the iMPScore values separating the cluster from each other. Once the cluster has been fixed, each participant has been assigned to one of the 5 clusters based on its individual iMPScore and the assignment has been set against the assignment to a MS category by an experienced therapist.

### Declaration of generative AI and AI-assisted technologies in the writing process

GPT-4 was used for language improvements. The authors reviewed and edited the content as needed and take full responsibility for the content of the publication.

### Ethical approval

The institutional ethics committee of our institution (EK254/16) granted approval for the study. Every participant’s parents / legal guardians gave their written informed consent prior to the evaluation. All methods were carried out in accordance with relevant guidelines and regulations of the institutional review board at the University Hospital RWTH Aachen and in accordance with the Declaration of Helsinki.

## Results

All participants were able to perform all three movement tasks and the acceleration signals from all sensors could be recorded. The only exception was the [HS] task, during which an artifact occurred in one of the sensors in two patients. For all three movement tasks, more than 6 features were identified with a linear correlation coefficient higher than 0.3, so that the slope coefficient of the regression line was decisive for the identification of the relevant features. The relevant features identified, and the classification results achieved with these features are presented separately for each of the three movement tasks below.

### Hand-to-mouth task [HM]

Table 1 lists the 6 features, including the sensors and the coordinates from which they were calculated, which were recognized as relevant by the feature extraction algorithm. It is noticeable that the chest sensor is particularly sensitive to pathological changes in the execution of a hand-to-mouth task. This is remarkable in that a single sensor has such high informative value, which, moreover, was not even localized on one of the extremities.

**Table 1:** The 6 features identified as relevant for the hand-to-mouth movement task based on a data set of 30 subjects each one performing 24 receptions of the HM task.

Hand-to-Mouth Task (*n* = 30)				
Feature	Sensor	Coordinate	Linear correlation coefficient	Absolute |S_i_| of the slope coefficient value
*Diff*_*Max*_	Chest	Z-axis	0.6	0.16
*Area*_*out*_	Chest	Z-axis	0.5	0.15
*Area*_*out*_	Chest	Y-axis	0.5	0.13
*CC*_*Coef*_	Chest	X-axis	0.5	0.13
*Diff*_*Max*_	Hand	Y-axis	0.5	0.13
*Area*_*out*_	Arm	Azimuth	0.3	0.09

Table 2 shows how the individual subjects are distributed across both the MS categories assigned by an experienced therapist and the clusters A to E, assigned based on the iMPScore. The median iMPScore is comparable between the MS categories, into which an experienced therapist has categorized the subject’s HM movement, and the clusters. In both the median of the iMPScore increases with increasing movement skills.

**Table 2:** Membership of subjects assigned to each MS category and the median iMPScore corresponding to the subjects in each MS category. Additionally, number of subjects assigned to a cluster and the median of iMPScore of each cluster including the cluster boundaries.

Hand-to-Mouth Task (*n* = 30)							
MS category	Number of subjects in the MS category	Median of the iMPScores in each MS category	Cluster	Number of subjects in each cluster	Median of the iMPScores of each cluster	Lower threshold	Upper threshold
*V*	10	73.9	*A*	10	72.3	68	100
*VI*	8	63.8	*B*	7	64.2	56	67
*III*	5	51.8	*C*	6	51.7	48	55
*II*	2	44.0	*D*	2	44.3	42	47
*I*	5	38.5	*E*	5	39.2	0	41

**Fig. 4:**
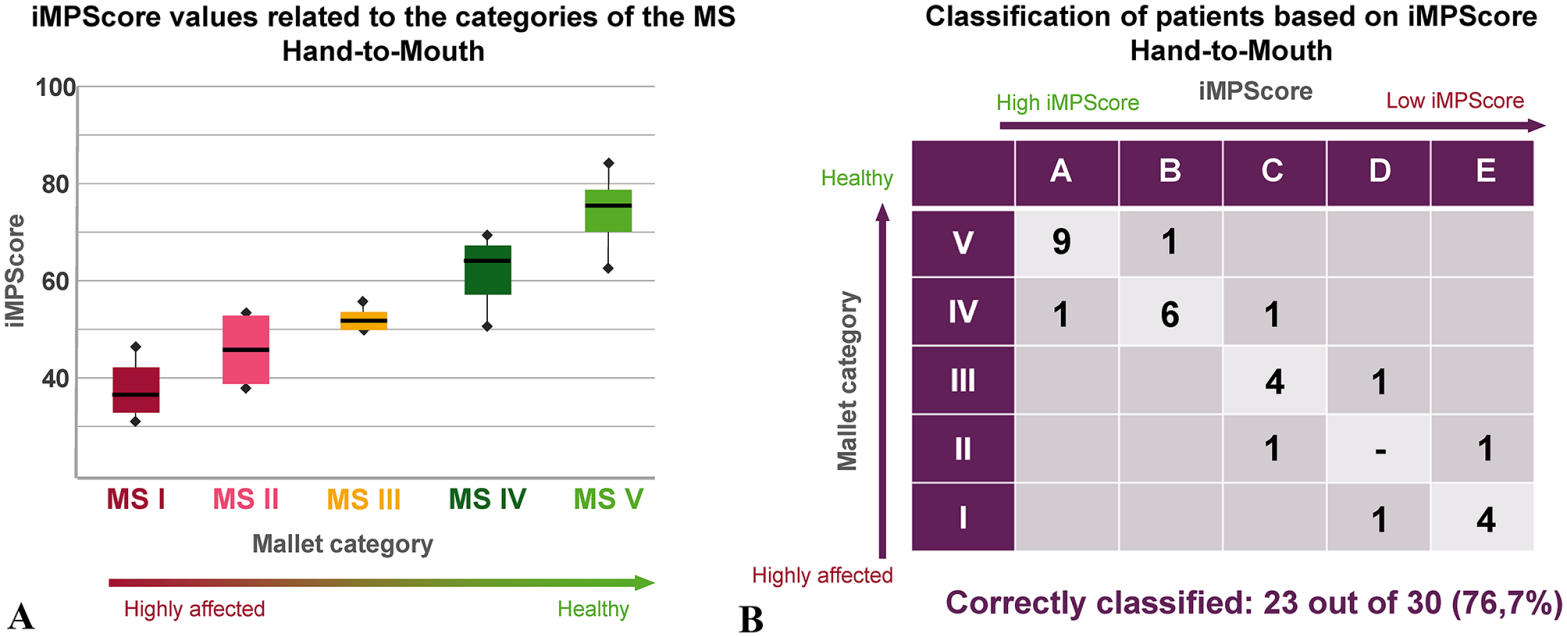
(**A**) correlation between the iMPScore determined using the patient-specific values of the relevant features and the MS category into which an experienced therapist has categorized the subject’s HM movement. (**B**) Classification results when grouping the subjects into 5 clusters using the iMPScore and a k-means algorithm.

Figure 4 A illustrates the correlation between the individual iMPScores and the MS category. The Classification results from grouping the participants into 5 clusters, based on the iMPScore and applying a k-means classification procedure as shown in Fig. 4B. Relevant thresholds between the clusters are summarized in Table 2.

23 out of 30 subjects were correctly clustered based on their iMPScore corresponding to their MS category for HM movement. The movement performance of a total of 3 patients (one out of 8 of MS IV, one out of 5 of MS III, one out of 2 of MS II) and one healthy participant were rated too poor by the algorithm, while 3 patients (one out of 8 of MS IV, one out of 2 of MS II) were rated too skilled. Misclassifications were made regardless of the degree of movement impairment (Fig. 4B).

### Hand-to-neck task [HN]

For the hand-to-neck movements, 6 features were identified as relevant (Table 3). In contrast to the hand-to-mouth movement, there is no preference for a sensor in the hand-to-neck task. In addition, the correlation with the corresponding MS category tends to be lower when performing a HN movement.

**Table 3:** The 6 features identified as relevant for the hand-to-neck movement task based on a data set of 30 subjects each one performing 24 receptions of the HN task.

Hand-to-Neck Task (*n* = 30)				
Feature	Sensor	Coordinate	Linear correlation coefficient	Absolute |S_i_| of the slope coefficient value
*Diff*_*Max*_	Hand	Elevation	0.6	0.17
*Diff*_*Max*_	Hand	X-axis	0.5	0.13
*Area*_*out*_	Chest	Z-axis	0.4	0.10
*CC*_*Coef*_	Arm	Azimuth	0.4	0.11
*Diff*_*Max*_	Chest	Z-axis	0.3	0.10
*Diff*_*Max*_	Arm	Azimuth	0.3	0.12

**Fig. 5:**
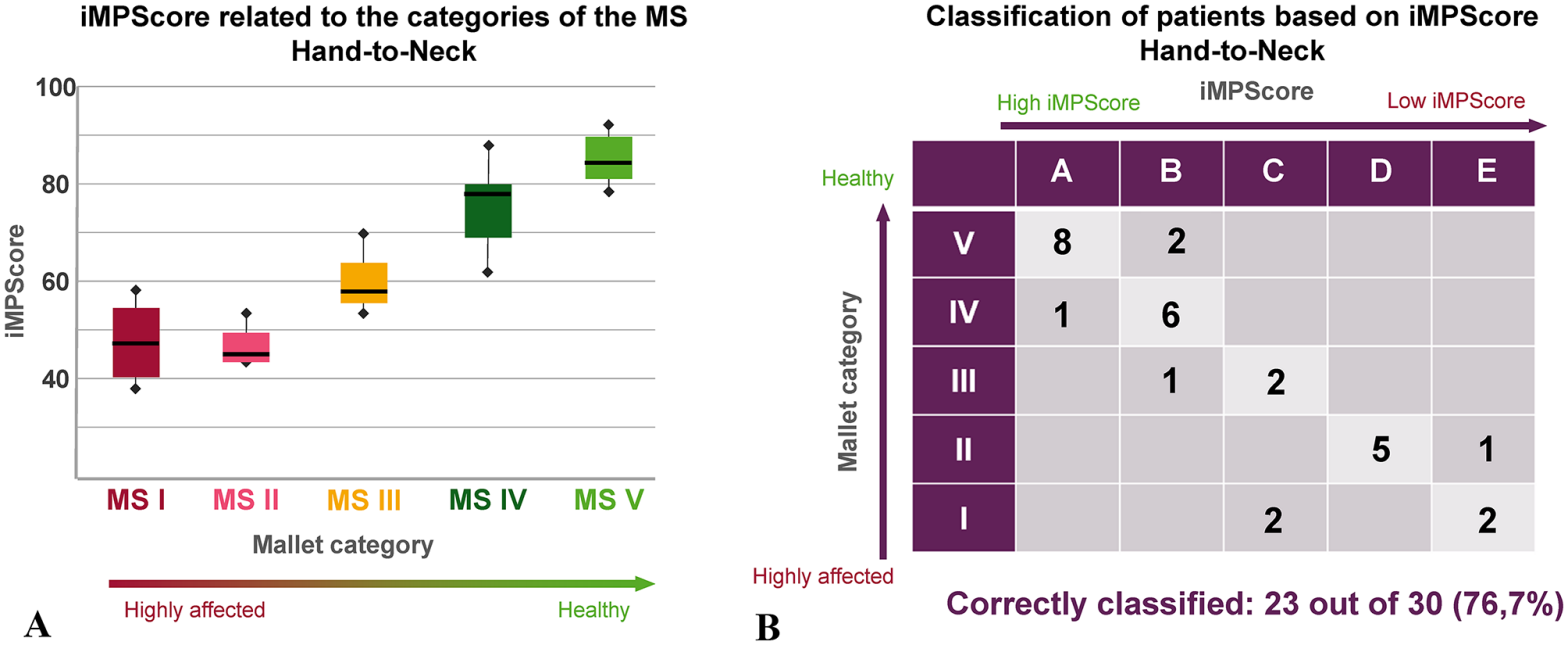
(**A**) Correlation between the iMPScore determined using the patient-specific values of the relevant features and the MS category into which an experienced therapist has categorized the subject’s HN movement. (**B**) Classification results when grouping the subjects into 5 clusters using the iMPScore and a k-means algorithm.

The distribution of the individual subjects across the MS categories and the individual clusters when performing a HN task is summarized in Table 4. As with the HM task, the median values of the iMPScore, when compared to the MS categories, are comparable to those when comparing to the clusters. Both increase with increasing functionality of the arm when an HN task is executed. The only exception are patients of MS category I, in which higher iMPScore values occur than in MS category II (see also Fig. 5A).

**Table 4:** Membership of subjects assigned to each MS category and the median iMPScore corresponding to the subjects in each MS category. Additionally, number of subjects assigned to a cluster and the median of iMPScore of each cluster including the cluster boundaries.

Hand-to-Neck Task (*n* = 30)							
MS category	Number of subjects in the MS category	Median of the iMPScores in each MS category	Cluster	Number of subjects in each cluster	Median of the iMPScores of each cluster	Lower threshold	Upper threshold
*V*	10	84.6	*A*	9	82.3	82	100
*VI*	7	79.1	*B*	9	75.3	66	81
*III*	3	56.2	*C*	4	55,4	51	65
*II*	6	44.3	*D*	5	45.1	43	50
*I*	4	46.9	*E*	3	38.2	0	42

Figure 5 summarizes the results for the hand-to-neck task graphically. As before with the HM task, 23 out of 30 subjects were clustered correctly based on their iMPScore for HN movements. One patient out of 6 of MS II and 2 healthy participants out of 10 were rated by the algorithm to have too poor movement skills, while 4 patients (1 out of 7 MS IV, 1 out of 3 MS II and 2 out of 4 MS I) were rated too skilled (Fig. 5B). As could be expected from the median iMPScores, the most serious misclassifications occurred in MS I patients. Half of them were assigned to cluster C, which corresponds to moderate arm function.

### Hand-to-spine task [HS]

Features characterizing the hand-to-spine task best are listed in Table 5. As with the HN movement task, information from the hand, upper arm and chest is required to recognize pathological movements during the HS task. However, the best correlation between the feature values and the assignment to the MS category is achieved for the hand sensor.

**Table 5:** The 6 features identified as relevant for the hand-to-spine movement task based on a data set of 28 subjects each one performing 24 receptions of the HS task.

Hand-to-Spine Task (*n* = 28)				
Feature	Sensor	Coordinate	Linear correlation coefficient	Absolute |S_i_| of the slope coefficient value
*Area*_*out*_	Hand	Y-axis	0.6	0.15
*Diff*_*Max*_	Hand	Y-axis	0.5	0.15
*Diff*_*Max*_	Hand	Elevation	0.5	0.15
*Area*_*out*_	Arm	Y-axis	0.5	0.14
*Area*_*out*_	Chest	Z-axis	0.4	0.11
*Area*_*out*_	Chest	Y-axis	0.3	0.12

**Fig. 6:**
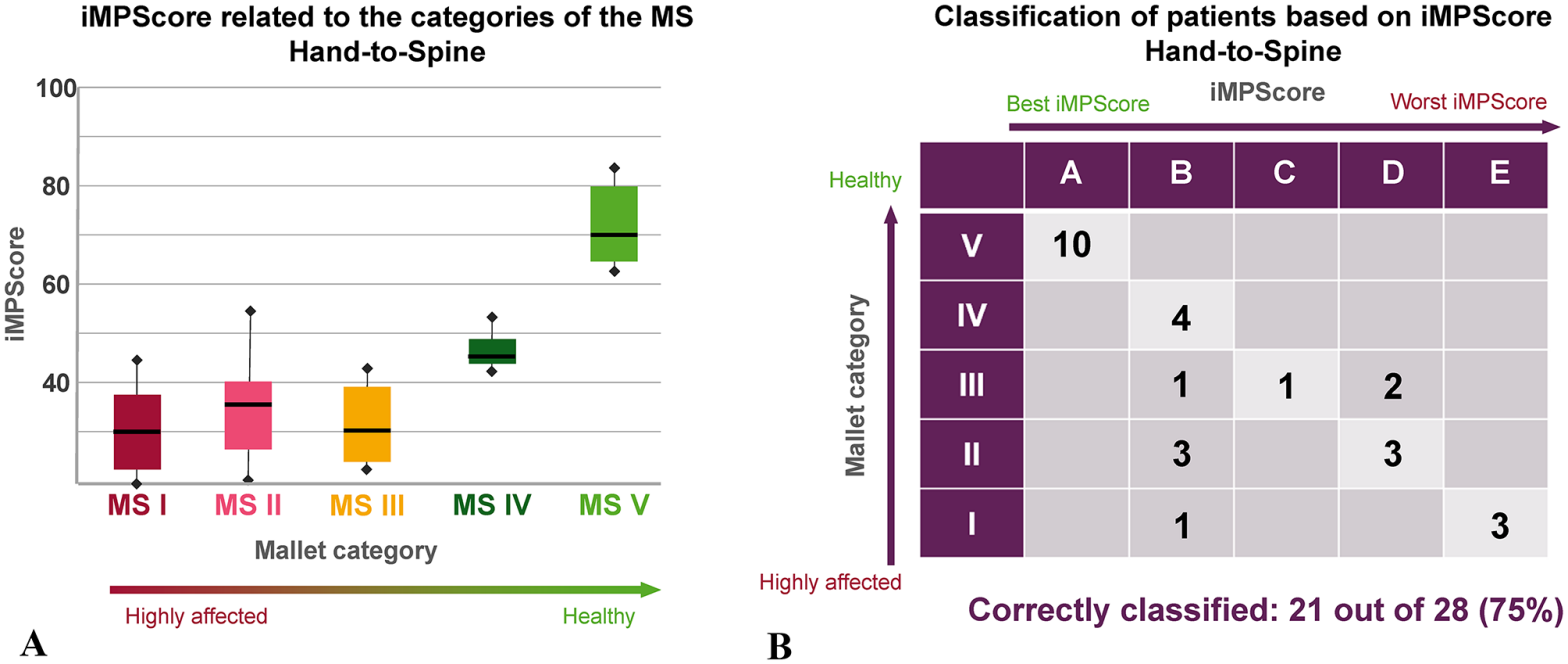
(**A**) Correlation between the iMPScore determined using the patient-specific values of the relevant features and the MS category into which an experienced therapist has categorized the subject’s HS movement. (**B**) Classification results when grouping the subjects into 5 clusters using the iMPScore and a k-means algorithm.

Table 6 shows that the median values of the iMPScores of patients in MS categories I to III hardly differ, while the iMPScores of patients in MS categories are higher when performing a HS task. This is also illustrated by Fig. 6A, in which there is no significant difference in the iMPScores of patients in MS I to III.

**Table 6:** Membership of subjects assigned to each MS category and the median iMPScore corresponding to the subjects in each MS category. Additionally, number of subjects assigned to a cluster and the median of iMPScore of each cluster including the cluster boundaries.

Hand-to-Neck Task (*n* = 28)							
MS category	Number of subjects in the MS category	Median of the iMPScores in each MS category	Cluster	Number of subjects in each cluster	Median of the iMPScores of each cluster	Lower threshold	Upper threshold
*V*	10	71.5	*A*	10	82.3	59	100
*VI*	4	43.8	*B*	9	75.3	39	58
*III*	4	31.9	*C*	1	55,4	35	38
*II*	6	35.9	*D*	5	45.1	34	34
*I*	4	29.2	*E*	3	38.2	0	33

Due to the fact that the iMPScores of patients in MS categories I to III hardly differ when an HS task is performed, the thresholds of clusters C to E are also very close to each other (Table 6). As a result, most of the misclassifications are made in patients of MS categories I to III (Fig. 6B). Overall, 21 of the 28 participants were correctly classified by the algorithm. However, these are mainly healthy subjects and patients in MS IV who have only minor movement impairments. Only 2 patients with MS III were assessed as having too poor movement ability. The functionality of the arm during the HS task is classified by the algorithm as too good in 5 patients with MS I to III. All these misclassified patients are assigned to cluster B corresponding to good arm function.

## Discussion

The primary aim of this study was to evaluate the consistency between accelerometer signal classification and the MS clinical test results in patients with OBPP and to validate the use of accelerometer data as a reliable tool for assessing upper extremity movement performance. Numerous studies have investigated upper limb function in post-stroke patients using accelerometers^18–21^. However, the use of accelerometers in the assessment of brachial plexus palsy is less explored^7,22,23^.

Our findings indicate a 75–77% consistency between accelerometer signal classification and MS clinical test results, which aligns with prior research^12^, supporting the validity of accelerometry in clinical assessments of OBPP. Despite the overall good performance of the accelerometers, some shortcomings were found. The iMPScore accurately identified subjects for the hand-to-mouth task. However, it failed to correctly classify the MS I group for the hand-to-neck task. As MS I signifies an absence of functional movement, there is no acceleration and, consequently, no discernible acceleration signal. This results in a very low signal-to-noise ratio, significantly increasing the likelihood of error in the readings. In addition, half of these patients were incorrectly classified, as they even didn’t attempt the task knowing they couldn’t perform it. By excluding the MS I group and adjusting the Class D range from 0 to 50 points for this task, the accuracy rate could be increased from 77 to 85%.

The iMPScore also struggled to accurately classify the MS I and II groups for the hand-to-spine task. Misclassifications occur as some patients are unable to complete the task. In addition, we suspect that the poor classification of the low scores could be attributed to the limited possibility of compensatory movements in the hand-to-spine action, which might allow the achievement of the desired position despite compromised functionality. Furthermore, the variations in arm posture across different MS categories for this movement are quite subtle, unlike other movements. Consequently, this leads to only minor differences in the acceleration signals, making it challenging to distinguish between categories. By modifying the Class C range to a span from 0 to 38 points and omitting the non-functional groups (MS I and II), the precision of this task could be enhanced from 64 to 94%.

However, the agreement between accelerometry and MS clinical test results underscores the potential of accelerometers to provide objective, quantitative assessments of upper limb function. This can probably enhance clinical evaluations, allowing for more precise monitoring of patient progress and tailored treatment plans^2,8^.

While traditional observational assessments like the Mallet Scale have their merits, including ease of use and cost-effectiveness, they are subjective and can vary between observers^8^. Accelerometry may offer a more standardized and objective alternative, reducing interobserver variability and providing detailed feedback on movement quality^23^.

The MS summarizes several task achievements into a “one class” belonging, thus being rather approximative. Some authors score the patient by adapting the MS to each task, thus offering five values for one assessment. Others add those 5 values to a sum from 5 (a minimum of 5 times value 1) to 25 (a maximum of 5 times 5). Both strategies generate a result which does not allow any feed-back to the range of limitation^2^. While the classification into MS categories is performed by a skilled therapist or surgeon, the process is inherently subjective^8^. This aspect becomes especially noteworthy when a patient falls at the borderline between two MS categories. Under such circumstances, the algorithm might arrive at a different conclusion than the therapist, suggesting that a deviation by one cluster in the iMP score could, in fact, be the correct decision, potentially indicating an error in the therapist’s judgment.

All features used for classification describe the difference in movement execution with the affected arm compared to the unaffected arm. On the one hand, these differences result from the fact that the movement task was not executed completely, as measured by the MS. However, differences in movement execution are also a result of compensatory movements that patients perform in order to fulfill the movement task as well as possible. Since, unlike MS, the acceleration signals are recorded over the entire period of movement execution, it can be assumed that compensatory movements contribute more to the feature values ​​than the fact that the movement task was not fully executed. Additionally, from clinical examinations, it is known that many compensatory movements are associated with a movement of the trunk. This clinical observation is reflected in feature values extracted from the chest sensor, which is mainly involved in the correct classification of patients. In addition, movements of the trunk influence the acceleration signals of the upper arm and hand. Consequently, with the introduced approach the entire posture of the upper body is analyzed during the execution of the task and compensatory movements in particular contribute significantly to the posture. It can therefore be concluded, that the ability to record and even to quantify the amount of compensatory movements is a key advantage of the presented approach, compared to the MS commonly used in clinical routine.

The fact that compensatory movements can be qualitatively analyzed allows for a more accurate comparison of outcomes over time, such as before and after surgery, during physical and/or occupational therapy, or throughout a rehabilitation program. The iMPScore could serve as a valuable measure to offer patients, doctors, and therapists immediate feedback on the quality of movement execution. In contrast, the Mallet Scale provides only a limited assessment of movement quality, focusing solely on the final position achieved without considering the quality of movement during execution^2^. Consequently, the iMPScore could allow for a more precise and comprehensive evaluation, capturing nuances in movement execution that the Mallet Scale may overlook. Even when patients do not progress in their Mallet Class, they could observe improvements through a higher iMPScore because of the finer graduation, offering encouraging feedback.

### Challenges and limitations

Assessing the movement quality of young patients remains a complex task, and the proposed method may not be suitable for all OBPP patients, especially those unable to understand the assessment process. However, this limitation may also apply to using the MS, which in general is also only validated and recommended for patients over the age of 2 being able to perform the 5 different motion tasks, respectively^4^. Additionally, the variability in patient numbers across task-groups within each MS category may have influenced the reliability and comparability among categories. However, the database size is quite limited - for certain tasks, there are as few as 2 patients represented in a specific MS category. Consequently, future studies with a larger patient sample are suggested to validate the iMPScore as an effective method for evaluating upper extremity movement quality with greater certainty.

In addition, one could assume that the variability in patient numbers across task-groups within each MS category may have influenced the reliability and comparability among categories. Specifically, as noted, for certain tasks, there are only few patients represented in a specific MS category. However, we took this fact into account when developing the classification procedure. According to Nieman^24^, a classification procedure is always meaningful if the number of features does not exceed the square root of the number of samples (patients). This is independent of the number of elements per cluster. Since we included 30 patients, we limited the number of features to 6 in order to achieve statistical significance.

### Clinical implications and future prospects

While accelerometers and Inertial Measurement Units (IMUs) are increasingly utilized in various clinical and research contexts to assess movement^7,23,25–27^, our study introduces a unique contribution to the field. The innovation of our research lies in the specific features extracted from the acceleration signals, providing a novel quantitative description of movement.

The application of motion analysis via accelerometers offers promising enhancements to clinical practice, providing valuable feedback to patients, doctors, and therapists. We are optimistic that with further development, this user-friendly method could become an integral part of daily clinical routines, enhancing patient care and treatment outcomes. Given the commonalities in movement patterns observed across various upper limb movement disorders, this method holds promise for broad applicability across a spectrum of conditions^13,19,22,23,26^. Future research, however, is necessary to confirm the efficacy of this approach.

## Data Availability

The datasets used and/or analyzed during the current study are available from the Department of Rehabilitation & Prevention Engineering, Institute of Applied Medical Engineering, RWTH Aachen on request (disselhorst-klug@ame.rwth-aachen.de).
